# An update on the advances in the field of nanostructured drug delivery systems for a variety of orthopedic applications

**DOI:** 10.1080/10717544.2023.2241667

**Published:** 2023-12-01

**Authors:** Wenqing Liang, Chao Zhou, Songtao Jin, Lifeng Fu, Hengjian Zhang, Xiaogang Huang, Hengguo Long, Wenyi Ming, Jiayi Zhao

**Affiliations:** a Department of Orthopedics, Zhoushan Hospital of Traditional Chinese Medicine Affiliated to Zhejiang Chinese Medical University, Zhoushan, China; b Department of Orthopedics, Zhoushan Guanghua Hospital, Zhoushan, China; c Department of Orthopedics, Shaoxing People’s Hospital, Shaoxing, China; d Department of Orthopedics, Shaoxing City Keqiao District Hospital of traditional Chinese Medicine, Shaoxing, China

**Keywords:** Nanotechnology, drug delivery system, orthopedic, bone, tumor

## Abstract

Nanotechnology has made significant progress in various fields, including medicine, in recent times. The application of nanotechnology in drug delivery has sparked a lot of research interest, especially due to its potential to revolutionize the field. Researchers have been working on developing nanomaterials with distinctive characteristics that can be utilized in the improvement of drug delivery systems (DDS) for the local, targeted, and sustained release of drugs. This approach has shown great potential in managing diseases more effectively with reduced toxicity. In the medical field of orthopedics, the use of nanotechnology is also being explored, and there is extensive research being conducted to determine its potential benefits in treatment, diagnostics, and research. Specifically, nanophase drug delivery is a promising technique that has demonstrated the capability of delivering medications on a nanoscale for various orthopedic applications. In this article, we will explore current advancements in the area of nanostructured DDS for orthopedic use.

## Introduction

Nanotechnology is a multidisciplinary scientific area that involves manipulating and controlling matter at the nanoscale size to make new materials, structures, and instruments. Feynman (2018) first presented the idea of nanotechnology in 1959. Since then, nanotechnology has become a promising area of research for many facets of human existence, such as transportation, electronics, the food industry, agriculture, energy, communications, medicine, and the biological sciences. The use of nanotechnology in the area of medicine, known as nanomedicine, can have a major influence on human health and has already altered and transformed various features of clinical study and practice. Material with specific chemical, physical, and biological features developed from nanotechnology provides a variety of novel options for therapeutic practices ranging from the prevention of diseases to therapy and diagnostics (Güven, 2020; Li & Yan, 2020; Mamun & Yuce, 2020; Cacciatore et al., 2021). Among all the medical uses of nanotechnology, nano-based drug delivery systems (DDS) have garnered the most interest in the study due to their high translational significance (Alp et al., 2019; Asil et al., 2020). Due to the accelerated advancements in nanotechnology, controlled medication delivery studies have achieved incredible advances. Nano-DDS offer a novel technique of medication administration for the cure of a variety of ailments and exhibit several benefits over conventional drug delivery methods. The nanoscale manipulation permits certain areas targeting and transport of pharmaceuticals, genes, and imaging agents, as well as their controlled release (Shi et al., 2010; Kavaz et al., 2010). Recent research has resulted in the advancement of numerous nano-based DDS made from diverse substances, including polymers, lipids, metals, small molecules, and inorganic materials (Patra et al., 2018). Orthopedics is one of the most prominent sectors within the vast array of nanotechnology-based drug delivery applications.

Nanotechnology has influenced and transformed every element of orthopedic research and practice over the past few decades. Due to its unique capability to produce materials and gadgets with exceptional mechanical, physicochemical, and biological characteristics, nanotechnology offers enormous potential in orthopedic applications (Kumar et al., 2020). It has been used in a number of innovative methods, for example, tissue engineering for the regeneration of bone, surface modification of implants and prostheses, targeted drug delivery, and orthopedic diagnostics (Yun et al., 2009; Parizek et al., 2012; Alp et al., 2017; Sun et al., 2018; Raina et al., 2020). Particularly, medication delivery systems based on nanotechnology have impacted numerous areas of orthopedics. In this article, we discuss the current advancement in nanotechnology-based DDS for orthopedics.

**Figure 1.**
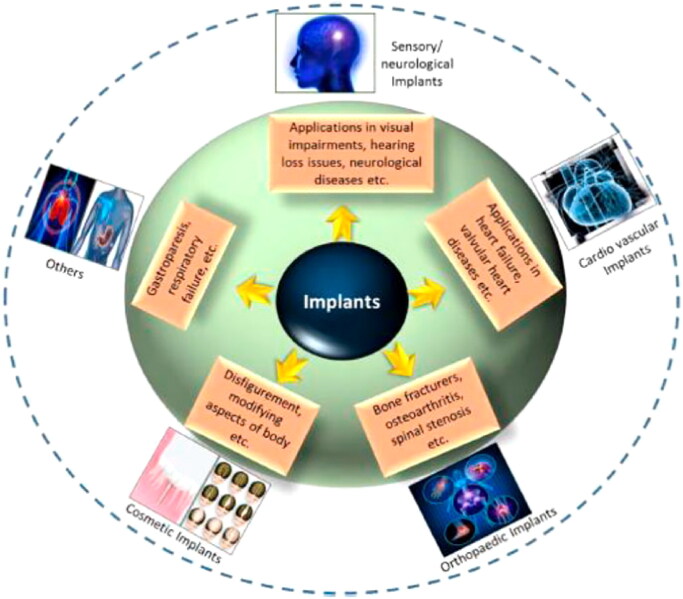
Implants are categorized according to their intended use, including cardiac grafts, orthopedic implants, cosmetic implants, neurological/sensory implants, and other uses offered. Using conventional metallic/nonmetallic materials, various engineering techniques have been found to imitate physical characteristics, chemical features, and gradient architecture of tissue or organs. Reproduced with permission from (Kumar et al., 2020).

As the population is aging, lifestyle modifications (specifically those that induce and extend chronic conditions like osteoarthritis and heart problems), bioengineering technological improvements, as well as enhanced public knowledge of cosmetic implants all help the exponential development of the bioimplant industry. As per market analysis, the worldwide bioimplant market is anticipated to reach $115.8 billion by the end of 2020, increasing at a compound annual growth rate (CAGR) of 10.3% over the projected period (2014–2020) (Zhao et al., 2021). Implants have developed as a possible revolutionary therapeutic choice for blindness, neurological problems, orthopedic concerns, cardiac problems, deformities, and dental deformities (Figure 1) (Ghezzi et al., 2013; Jackson, 2016; Scaini & Ballerini, 2018). Various implants, that includes replacements for joints, sutures, vascular implants, bone plates, valves of the heart, ligaments, dental grafts, intraocular lenses and several others, are frequently utilized to (i) replacement or restoration of function of damaged or disintegrating tissues, (ii) alter the functionality of a physical component, (iii) promote restoration, and (iv) repair defects for cosmetic reasons. Utilizing conventional metallic/nonmetallic materials, Various engineering strategies for mimicking the physical characteristics, chemical features, and gradient architecture of tissues or organs have been reported (Cross et al., 2016). Several engineering techniques employ typical metallic materials that are not metallic to simulate the chemical and physical characteristics of other materials, and tissue or organ gradient architecture. Though, conventional implants have many drawbacks. They rarely react with tissues, are unsuitable for use with tissues, and are sometimes not supported by the human body (Bian et al., 2016). In recent years, nanotechnology’s impact on the graft market has increased. Particularly, researchers are looking at the possibility of nanomaterials with biologically inspired qualities for enhancing the functionality of conventional grafts. This study looks at the creation of biomaterials for orthopedic use, from conventional (i.e. nonmetallic and metallic) ingredients for NPs. Orthopedic therapies rely largely on a precise diagnosis of therapeutic sites and successful embedding. Recent advances in essential orthopedic biomaterials, such as smart biomaterial, nanocomposite grafts in 3D printed form, and porous substances, as well as commercial issues, are addressed to offer a complete understanding of this rapidly growing scientific discipline. This research offers a strong basis for incorporating nanotechnology-powered orthopedic implants into the body of humans.

## Nano-based DDS application in orthopedics

DDSs are designed structures that are employed for transportation and safeguarding, and control of the discharge of a pharmaceutical compound. Methods for delivering drugs using nanotechnology offer various benefits over conventional drugs, for example, a high surface area-to-volume ratio for effective loading of the drug, better targeting as a result of their tiny size, which enables them to pass through biological impediments, enhanced biological medium physical stability and drug solubility, and the feasibility of functionalization surface for sensation, imaging, diagnosis, and the attachment of biologically targeted moieties the administration of medicines (Chen et al., 2019; Yang et al., 2020; Zhou et al., 2020).

Lipid NPs, polymeric NPs, quantum dots, dendrimers, metallic NPs, and carbon nanotubes are only a few instances of nanotechnology-based DDS that have significantly advanced the area of drug delivery and the medical sector as a whole (Lu et al., 2010; Bhirde et al., 2010; Soiberman et al., 2017; Aizik et al., 2019; Dave et al., 2020) Various delivery techniques have been used to cure bone-related diseases to increase the effectiveness and specificity of traditional therapeutic therapy. Many bone-related disorders, for instance, osteosarcoma, osteoarthritis, osteoporosis, cancer bone metastases, infection of the bone, and inflammatory disorders, along with bone tissue healing and renewal, have been treated with NPs and nano-based scaffolds (Jin et al., 2020; Mao et al., 2020; Murthy et al., 2020; Li et al., 2020; Hassani Besheli et al., 2017). Some of the DDSs for the application in the field of orthopedics are summarized in Table 1.

**Table 1.** Application of various drug delivery systems in the field of orthopedics.

	Carrier	Cargo	Outcome	Ref
Liposomes	(HA)-liposomal	Dexamethasone, Diclofenac,	Significantly alleviating OA pain and being biocompatible	Chang et al. (2021)
	SLN system	pDNA for integrin *β*1 overexpression	Reduced chondrocyte apoptosis and improved tissue healing	Zhao et al. (2020)
	hollow zoledronic acid-contained nanoparticles	ZOL and Ca^2+^	A Precisely Controllable Bone-Penetrating Drug Release System Allows Localized Therapy of Osteoporotic Fracture Prevention by Modulating Osteoblast-Osteoclast Communication	Liang et al. (2023)
Micelles	Polyethylene oxide- (PEO-) and polypropylene oxide- (PPO-) based polymeric micelles	rAAV sox9	Increasing ECM components accumulation and cell survival while decreasing inflammation	Urich et al. (2020)
	tetracycline (TC)-grafted methoxy poly-(ethylene-glycol)-poly-(D, L-lactic-co-glycolic acid) (mPEG-PLGA) micelles (TC-mPEG-PLGA) with TC and mPEG-PLGA	Astragaloside IV	The nano drug delivery system (TC‒mPEG‒PLGA) could target bone *in vitro* and *in vivo*, whereby it might be employed as a new delivery technique to improve the therapeutic benefits of medicines with osteoporotic activity.	Que et al. (2022)
	PNIPAM-PMPC	Diclofenac sodium	PNIPAM-PMPC nanospheres are biocompatible and increase anabolic gene expression while decreasing articular cartilage catabolism gene expression.	Zhang et al. (2020)
	Acid-activatable polymer	Curcumin	Inhibition of tumor necrosis factor-alpha (TNF-*α*) and interleukin 1*β* (IL-1*β*). Strong antioxidant and anti-inflammatory properties.	Kang et al. (2020)
Exosomes		miR-140, miR-100-5p, miR-9-5p, miR-1405p, miR-135b, and lncRNA KLF3-AS1	Lowering inflammation and increasing the synthesis of cartilage markers	Liang et al. (2020); Tao et al. (2017); Liu et al. (2018)
		pTa	This new composite scaffold can successfully induce bone regeneration in locations with significant bone defects.	Yang et al. (2023)
				
Inorganic NPs	Membrane-disguised Fe_3_O_4_	Kartogenin	Accelerating and improving the regeneration of cartilage	Zhang et al. (2020)
	MSNs	Gold-based nanoformulations, MnO_2_, CeO_2_	The expression of the genes ACAN and COL2a1 in chondrocytes was dramatically reduced.	Chen et al. (2021); Famta et al. (2020)
	Zeolitic imidazolate framework-8	*S*-Methylisothiourea hemisulfate salt	Decreasing NO and H2O2 levels, therefore limiting the development of HIF1 and M1 macrophages and improving mitochondrial function.	Zhou et al. (2020)
Polymer NPs	LbL polymer microcapsule	MnO_2_	Reducing H2O2 and safeguarding cells against oxidative stress	Marin et al. (2020)
	Poly(D,L-lactic acid)-poly(ethylene glycol)-poly(D,L-lactic acid)	BMP2	causing graft differentiation into bone and cartilage while also being destroyed without toxicity	Wu et al. (2020)
	CD-PMPC	Silica	Increasing cutaneous tissue penetration and lubrication, as well as promoting medication release	Zhao et al. (2021)
	PLGA-PEG	4MAL, kartogenin (KGN)	Increasing the retention of IA drugs for the management of OA. enhancing the amount of sulfated glycosaminoglycans	Zerrillo et al. (2021)
	Electrostatic self-assembly heparin and *ε*-poly-l-lysine	Platelet lysate	The platelet lysate is dispersed equally	Tang et al. (2021)
	Electro-spun cell-free fibrous hyaluronic acid	SDF-1*α*, TGF-*β*3	Increased recruiting and MSC infiltration to improve cartilage tissue development	Martin et al. (2021)

Although the NP-based DDS exhibits considerable targeting potential in the treatment of OA, its clinical applicability is still limited. PNP particles, like PLGA, have been utilized extensively due to their varied forms. However, rejection by the immune system may result in an in vivo novel inflammatory response (Chen et al., 2023). Polymer NPs modified with HA, chitosan, or other substances represent a novel strategy for enhancing the biocompatibility of nanomaterials. Exosomes, on the other hand, have excellent biocompatibility as an endogenous substance, however, their progress is hampered by the purifying procedures and exceedingly poor yield. Increasing the yield of exosomes significantly is a critical area for upcoming exosome studies. Though liposome-based DDSs have been the subject of extensive research, they are not the optimal choice for hydrophilic pharmaceuticals. Very low targeting and insecurity constrain the further applications of inorganic nanoparticles (Chen et al., 2022). Bioceramic NPs are the best alternative for reparative and restorative bone treatment due to their biomimetic composition, bioactivity, and good assimilation into the natural bone structure. Their well-known cytocompatibility and favorable connections with living tissues can be examined in combination using polymeric constructs and inorganic (alloys, ions, NPs, and composites) or organic (biomolecules and drugs) materials to create bone-mimicking platforms for the particular and selective treatment of bone pathologies (Burdușel et al., 2022).

### Osteoarthritis

Osteoarthritis (OA) is one of the most prevalent joint disorders and is the main cause of impairment, and its prevalence is increasing globally. The existing therapeutic options for osteoarthritis are inadequate, and a large number of them concentrate mainly on pain alleviation as opposed to slowing the progression of the disease. Systemically delivered osteoarthritis medicines are hampered by speedy clearance following intra-articular injection, restricted cartilage targeting, and significant adverse consequences caused by numerous high-dose drug administrations. Nanotechnology-based DDSs have been studied to improve the pharmacokinetics and pharmacodynamics of pharmaceuticals for osteoarthritis through sustained and targeted curative impact with minimal systemic side effects and longer-term advantages (Sacchetti et al., 2014; Lin et al., 2016; McMasters et al., 2017; Yan et al., 2019; Rahmani Del Bakhshayesh et al., 2020).

**Figure 2.**
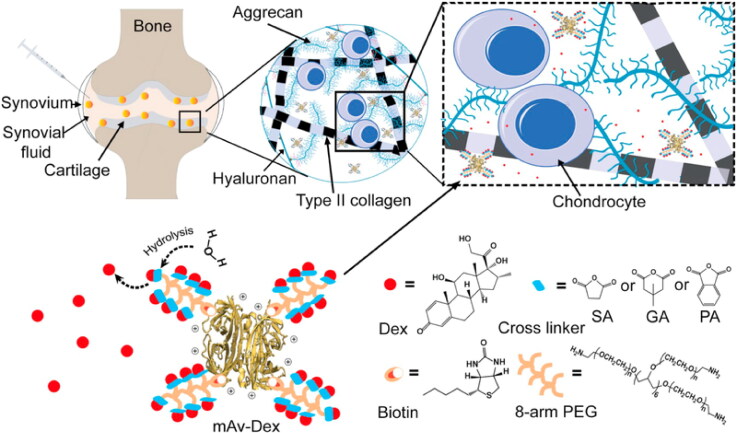
Charge-based intra-cartilage drug distribution of the nano-construct multi-arm Avidin coupled with a tiny molecule of a drug, Dexamethasone (mAv-Dex) by using hydrolyzable ester linkers derived from glutaric succinic, and phthalic anhydrides (SA, PA GA,) in 2:1:1 molar ratio permitting tunable (and long) drug release half-lives. Reproduced with permission from (He et al., 2020).

Maudens et al. (Johnson et al., 2012) made kartogenin nanocrystal-polymer particles as a controlled drug release technology for the efficient prevention of OA with a significant drug loading over a prolonged period. Kartogenin is a heterocyclic molecule that is tiny in size and capable of protecting and regenerating cartilage. In a murine model of mechanistic osteoarthritis, Kartogenin nanocrystal-loaded polymeric particles outperformed kartogenin solution in terms of bioactivity, demonstrating a unique as well as a novel extended DDS. In a research investigation by Kang et al., (2016). Nanospheres with thermoresponsive properties were made to release kartogenin and diclofenac simultaneously and independently in a unified system for coupled osteoarthritis treatment. Independently, the nanospheres released diclofenac first in an explosive manner and kartogenin over time in reaction to temperature variations. Experiments in vivo and in vitro indicated that these thermoresponsive nanospheres exhibit chondroprotective as well as anti-inflammatory properties in the cure of OA (Kang et al., 2016). He et al. (2020), also devised and reported a cationic multi-arm avidin nano-construct for intra-cartilage administration of a wide range of small molecule osteoarthritis medications and their interactions with chondrocytes. The objective is to create a nano-construct for charge-based intra-cartilage medication administration, which involves designing a multi-arm Avidin (mAv) with 28 sites available for drug covalent conjugation (Figure 2). Due to its diminutive size and optimum positive charge, avidin is able to easily enter into cartilage deeply (Bajpayee et al., 2015). As a DDS for the cartilage, an Avidin nano- construct might have the ability to provide a mixture of medications with a single injection, removing toxicity concerns and maintaining the release of the drug inside the joint for effective and secure osteoarthritis therapy (He et al., 2020). The charge-based intra-cartilage DDSs involve a nano-construct known as multi-arm Avidin coupled with Dexamethasone (mAv-Dex) small molecular drug. To provide adjustable and extended drug-release half-lives, this system employs hydrolyzable ester linkers generated from glutaric, succinic, and phthalic anhydrides in a 2:1:1 M ratio. Due to electrostatic connections, mAv-Dex may easily permeate the whole thickness of negative charge cartilage after intra-articular administration, forming an intra-cartilage drug depot. The mAv has an optimum net positive charge, allowing for quick and high intra-cartilage uptake as well as long-term retention through weak-reversible coupling with negative charge aggrecans. The recommended doses of Dexamethasone are then released over several days through hydrolysis from mAv, which can interact with the glucocorticoid receptors on chondrocytes and suppress the catabolic activity associated with osteoarthritis (OA). These DDSs can be employed to transport several drugs or drugs combination, allowing OA therapy with a single injection of low-dose drugs, which eliminates toxicity complications related with several high-dosage injections required to keep sustained drug doses throughout the joint (Figure 2) (He et al., 2020).

### Oncology in orthopedics

Osteosarcoma is the most prevalent malignant early bone cancer. Chemotherapy is an essential osteosarcoma therapy, however, it is hampered by significant adverse reactions and medication resistance (Steckiewicz & Inkielewicz-Stepniak, 2020). Nanotechnology advancements have resulted in the creation of multipurpose nanoparticles as diagnostic and treatment agents capable of specifically targeting bone tumor cells and delivering medicines and genes (Kılıçay et al., 2011; Zhang et al., 2018). Nanocarrier-mediated targeted delivery and controlled release of chemotherapeutic drugs prevents quick drug clearance, prolongs blood circulation time, and increase intra-tumoral deposition, boosting therapeutic effectiveness and minimizing side effects (Kılıçay et al., 2011).

Bone cancer-related skeletal complications are an essential health care issue. Osteosarcoma is the most typical type of bone tumor and the 3^rd^ biggest cause of cancer in children and adolescents (Kansara & Thomas, 2007). The most prevalent region of cancer metastases is bone and is especially significant in prostate and breast malignancies due to the high prevalence of bone metastases in these diseases (Ovid’ko & Sheinerman, 2011). The most usual methods for treating bone metastases are radiotherapy, surgery, bisphosphonates, systemic chemotherapy, and radioisotopes (Suva et al., 2011). The latest developments have resulted in the creation of multifunctional bionanomaterials capable of targeting and delivering therapeutic medications or DNA to bone tumors (Mohamed et al., 2012). To induce hyperthermia, a variety of composite materials having differing amounts of magnetite are utilized (Li et al., 2006; Hu et al., 2006). Andronescu et al. (2010) manufactured a bone implant component and hyperthermia generator made of magnetite-enriched collagen/HA composite for the cure of cancer of the bone. Hu et al. (2006), fabricated a 3D nanomagnetite/CS rod that might be used to induce hyperthermia locally in bone lesions. Murakami et al. (2008) created a magnetite/HA composite that allows for direct adherence to bones via HA as well as heat production from magnetite (when subjected to an AC magnetic field) for bone cancer hyperthermia therapy. This composite had microporous dimensions of almost 400 μm and submicroporous dimensions of about 0.2 μm. Magnetite collections were shown to be firmly confined in rod-shaped HA particle cages at concentrations of 30 mass percent or less.

**Figure 3.**
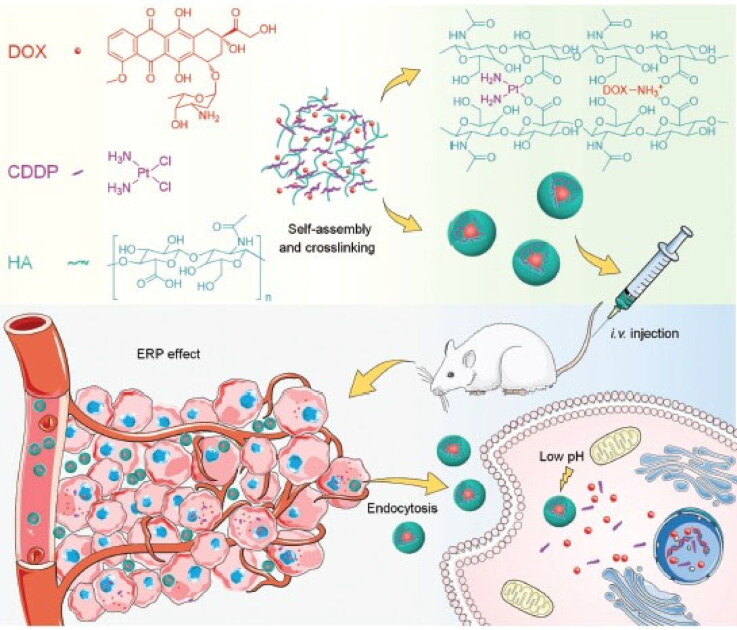
HA encapsulates CDDP and DOX through electrostatic and chelation connections having its carboxyl groups on the sides. Representation of preparations, parenteral administration, in vivo circulation, specific development in tumor tissue, and CDDPHANG/DOX intracellular release of drug-mediated by pH (Zhang et al., 2018).

It has been reported that a doxorubicin-loaded hyaluronic acid nanogel crosslinked with cisplatin effectively treats osteosarcoma (Zhang et al., 2018). This delivery system had a longer time of circulation than unbound medications due to the increased stability of the nanogel. Following valid tumor accumulation, apoptosis-inducing synergistic actions of cisplatin and doxorubicin were reported, indicating its tremendous potential for osteosarcoma chemotherapy. HA, one of the most intriguing biomaterials, was used to encapsulate CDDP and DOX through electrostatic and chelation connections having its carboxyl groups on the sides, correspondingly (Figure 3) (Zhang et al., 2018). In a separate study, Au et al. (2016) developed pH-responsive nanoscale metal-organic scaffolds for calcium zoledronate administration in people with cancer to cure bone metastases. Folate, a targeted ligand, was added to improve tumor nanoparticle (NPs) uptake. The improved tumor absorption and the medication release kinetics may account for the 80 to 85 percent rise in the direct anticancer activity of zoledronate as compared to the unbound drug, as demonstrated by the authors in vivo (Au et al., 2016). Moreover, an intelligent nano DDS comprising of graphene oxide function coupled with polyethylene glycol, folic acid, as well as the photosensitizer indocyanine green was designed for the administration of MutT homolog 1 blocker and doxorubicin. This nano-DDS with photothermal and photodynamic transformation capabilities inhibited osteosarcoma through a combination of chemo-photodynamic effects (Au et al., 2016).

### Osteoporosis

Osteoporosis is a progressive and degenerative bone disorder that affects hundreds of millions of elderly people globally, leading to a rise in bone fragility and fracture susceptibility (Wei et al., 2016; Bozkurt et al., 2019; Wang et al., 2020). Anti-osteoporotic medications administered systemically result in adverse effects because of off-target tissue impacts. Consequently, there is a need for novel pharmaceuticals with greater treatment effectiveness, fewer adverse side impacts as well as more efficient administrative ways. In contrast, nanostructured drug delivery systems, which can enable particular drug release kinetics, boost drug concentration at the local level, minimize side effects, and accelerate bone healing, are novel and alternative osteoporosis therapy techniques.

To repair osteoporotic local abnormalities, Brush-modified mesoporous hydroxyapatite NPs with simvastatin-loaded poly (N-isopropylacrylamide) were employed to create a controlled drug release system (Wu et al., 2020). As a novel strategy, this method provided sustained simvastatin release to prevent mesoporous hydroxyapatite and osteoporosis NPs to promote osteogenesis. As a bone-specific delivery method, Ryu et al. (2016), developed alendronate-conjugated nanodiamonds to achieve a synergistic impact in the cure of osteoporosis. Selective bone tissue formation, a strong affinity for hydroxyapatite, a good synergistic impact for the activity of alkaline phosphatase, and *in vivo* bone-targeted capability revealed the promise of osteoporosis treatment using alendronate-conjugated nanodiamonds. In a similar research, Nagai et al. developed transdermal preparations with raloxifene solid NPs to boost raloxifene’s limited bioavailability by utilizing a penetration promoter and tested them in an ovariectomized rat model for osteoporosis treatment (Nagai et al., 2018). In this investigation, a high rate of transdermal raloxifene skin permeation and significant osteoporosis therapeutic effects were recognized.

In preclinical studies, Pharmaceuticals with BP modifications have been developed to treat osteoporosis. The preparation of hormones that have been PEG-conjugated, for instance, parathyroid hormone (PTH) or calcitonin, and the synthesis of conjugates arbitrated by BP. In animal models, the impact of these hormones as mediated by BP was examined, and these bone-targeting medications demonstrated a substantial increase in bone strength and development in rodents with osteoporosis models (Bhandari et al., 2015). Also developed were BP-conjugated prodrugs of prostaglandin E2 and 17-estradiol (Tsushima et al., 2000). In animal models of osteoporosis, repeated treatment of any of these conjugated substances resulted in specific delivery to bone and an enhancement in BMD relative to free compounds.

Denosumab and teriparatide, the 02 most often utilized bio-derived osteoporosis treatments, are only available as subcutaneous injections (Jia et al., 2015). Denosumab is administered in 60 mg quantities twice yearly. Teriparatide, on the other hand, is recommended as 20 g daily subcutaneous injections. As is typical for injectable medications, the bioavailability of teriparatide and denosumab exceeds 90 percent.

Only estradiol and calcitonin are not typically administered orally or intravenously among currently approved osteoporosis treatments. Estradiol is offered as a film for transdermal administration, whereas calcitonin is available as a nasal aerosol for inhalation. Both therapies have the potential to take advantage of the comparatively high diffusive ability of each hormone to go through the epidermis and mucous membranes, correspondingly, relative to typical therapeutics (Posadowska et al., 2015). Estrogen’s steroid hormone status confers a high level of permeability within cells, permitting the bioavailability of transdermal to be almost 20 times greater than oral bioavailability (Posadowska et al., 2015). Likewise, calcitonin seems to be effectively endocytosed via nasal epithelial cells, resulting in an atypically large absorption capacity relative to its size (Farra et al., 2012).

**Figure 4.**
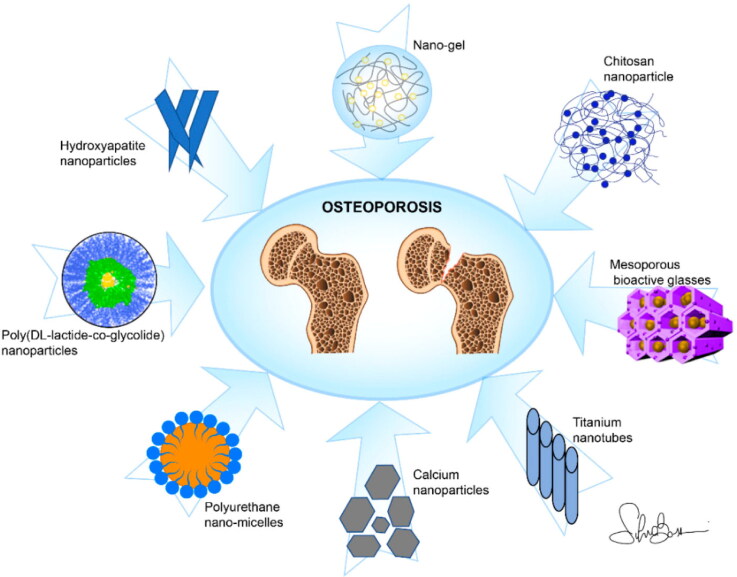
Various drug delivery systems against osteoporosis (Salamanna et al., 2021).

By either suppressing or activating osteoclasts, drug therapies for osteoporosis are designed to restore bone balance. Oral delivering bisphosphonates or hormones, and bisphosphonate intravenous infusion with teriparatide injection are all vulnerable to noncompliance of patients and low bioavailability. Transdermal administration of steroidal hormones and peptide hormone inhalation avoids these difficulties only partially. As novel modulators of bone differentiation of cells and activity, including antibody-mediated treatments or specific receptor agonists and antagonists, are discovered, enhanced ways of administering these bioactive molecules become increasingly important. Bone-targeting, encapsulation in or conjugation with biodegradable polymers, and both passively and actively activated depot systems can supply the drug where it is required, safeguard it from deterioration, and keep healing dosages whereas reducing compliance with patient difficulties (Asafo-Adjei et al., 2016). Figure 4 shows the various drug delivery systems against osteoporosis.

### Orthopedic infections

Nanotechnology has shown tremendous promise to cure and prevent bone infections by using antibacterial NPs to enhance antibiotic efficiency and eliminate resistant bacteria in certain circumstances. Antibiotic delivery systems based on nanotechnology have numerous benefits over free antibiotics, such as sustained and controlled kinetics of medication release, simplicity of surface modification for targeting bone or bacteria, a high level of local bioactivity, minimal systemic adverse effects, the capability to administer a variety of antibiotics, as well as increased medication stability and solubility (Guo et al., 2018; Qayoom et al., 2020).

Certain nanostructures, like metallic NPs, possess inherent antibacterial properties. Qadri et al. (2017), reported a prospective inorganic approach as an antibiotic-free and successful alternative therapy for osteomyelitis by employing silver-copper-boron composite NPs’ antibacterial activity to remove the infection with *Staphylococcus aureus* of the bone in a mouse model of osteomyelitis (Qadri et al., 2017).

**Figure 5.**
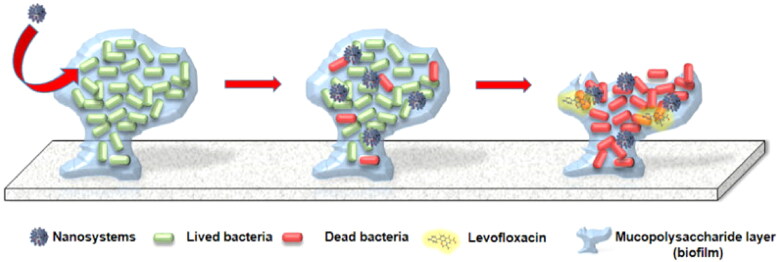
A novel nanoantibiotic has been developed, which comprises mesoporous silica nanoparticles (MSNs) loaded with the antibacterial ingredient LEVO and grafted with ConA outside the body. This design has been shown to selectively identify and attach to specific glycans. Reproduced with permission from (Martínez-Carmona et al., 2019).

As a new type of targeted delivery system (Figure 5), concanavalin A-coated mesoporous silica NPs laden with levoxacin were engineered. Concanavalin A covalent grafting to NPs increased the antibacterial efficacy of levofloxacin by facilitating its penetration into Gram-negative bacteria biofilm. Internalization of biofilm in conjunction with an antibacterial agent produced a significant antimicrobial action against bacterial biofilm (Martínez-Carmona et al., 2019). The use of antimicrobial NPs in conjunction with implants allows for simultaneous osseointegration and infection elimination (Song et al., 2017). Similar to antibacterial nanomaterials, bioactive matrix materials can be conjugated with antibacterial nanomaterials to form dual-functional composites that can both eliminate infection and serve as a framework for improved osteogenesis (Stravinskas et al., 2018; Wang et al., 2019). Combining scaffolds with a nano-based system for drug transport stands out as an effective approach for both enhancing tissue restoration and eliminating the infection.

Existing bone cements have several drawbacks, like poor antibacterial efficacy and antibiotic elution from the polymethylmethacrylate matrix (Atıcı et al., 2018). Many antibiotic delivery systems based on nanotechnology, comprising clay nanotubes, polymeric NPs, hydroxyapatite nanorods, liposomes, and carbon nanotubes, have been investigated to increase the medication release profile of polymethylmethacrylate bone cement (Al Thaher et al., 2017; Aşık et al., 2019; Çağlar et al., 2020).

### Bone regeneration

Despite recent advancements in surgical and pharmaceutical therapies, bone regeneration and reconstruction continue to be significant concerns in orthopedic drugs. Tissue engineering of bone is regarded as a potential technique for the restoration and renewal of bone tissue employing a wide range of bioactive components (Raina et al., 2020). Nanotechnologies seem to play a substantial and potential role in bone tissue engineering. By replicating biological micro- and nano-environments, scaffolds with nanostructures establish a perfect environment for cell propagation, adhesion, proliferation, and bone development, along with regulated drug and growth factor distribution to the lesion location. Additionally, NPs can be used with scaffolds to aid in bone repair.

**Figure 6.**
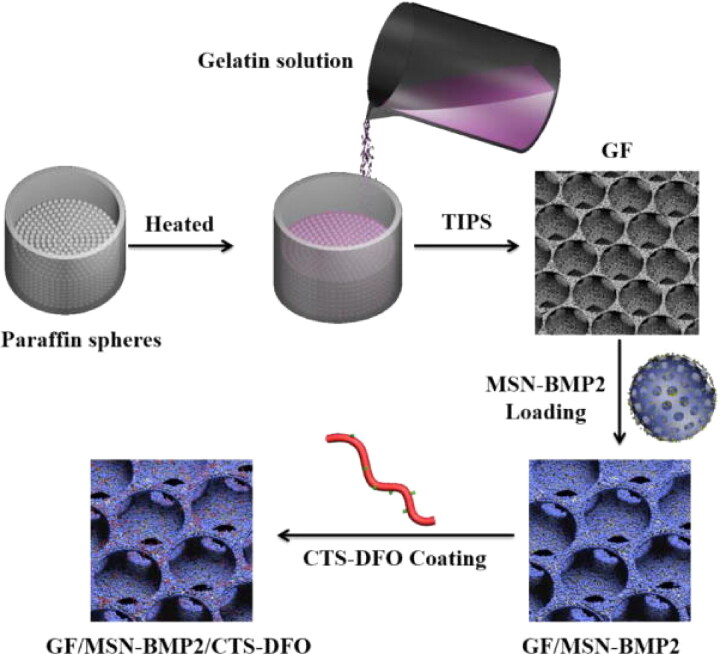
TIPS&P technique was used to fabricate GF scaffolds. Reproduced with permission from (Yao et al., 2018).

Numerous published research studies describe the utilization of drug-delivery nanostructured scaffolds for the regeneration of bone tissue (Hm et al., 2019; Cheng et al., 2019; Cheng et al., 2020; Limongi et al., 2020; Bhattarai et al., 2020). As a biomimetic osteogenic environment, A nanofibrous gelatin scaffold with mesoporous silicate NPs was created for the simultaneous administration of deferoxamine and bone morphogenic protein 2 (Yao et al., 2018). The results demonstrated that the created scaffold might regulate the simultaneous administration of bone morphogenic protein 2 and deferoxamine at different release rates, whereas retaining their angiogenic and osteogenic characteristics, correspondingly. Figure 6 illustrates the synthesis of the procedure of 3D GF scaffolds.

Figure 6 explains the fabrication of GF scaffolds. Briefly, at 37 °C, gelatin B was dissolved in a 50% ethanol/water solvent to produce a 7.5% gelatin solution. The gelatin solution was mixed in paraffin cylinders before they were transferred to an 80 °C freezer overnight to separate phases. The specimens were then submerged in ethanol at 20 °C for 24 hours. The specimens were then moved to a 1, 4-dioxane solution for an additional 24 hours. Finally, the materials were lyophilized after being frozen overnight at 20 °C for 48-hour salt-ice treatment. Using a biopsy tool (Premier Medical Product Company, PA, USA) and a blade, the scaffolds were cut into 5 mm x 2 mm disks. The gelatin-paraffin scaffolds are freeze-dried were subsequently submerged in hexane to extract the paraffin spheres. The specimens were frozen for 48 hours in the salt-ice immersion. Chemical crosslinking of 3D GF scaffolds was performed in a MES buffer (pH 5.3, 0.05 M) with NHS and EDC as the cross-linker for 24 hours at 4 °C. The GF scaffolds were rinsed with DI water and freeze-dried for 48 hours after crosslinking.

For the healing of bone fractures, a parenteral preparation of a hybrid NPs/hydrogel small interfering ribonucleic acid (siRNA) transference technique was formulated (Wang et al., 2017). The outcomes demonstrated that the formulated system substantially increased bone formation and accelerated recovery, making it a promising material for fracture curing.

**Figure 7.**
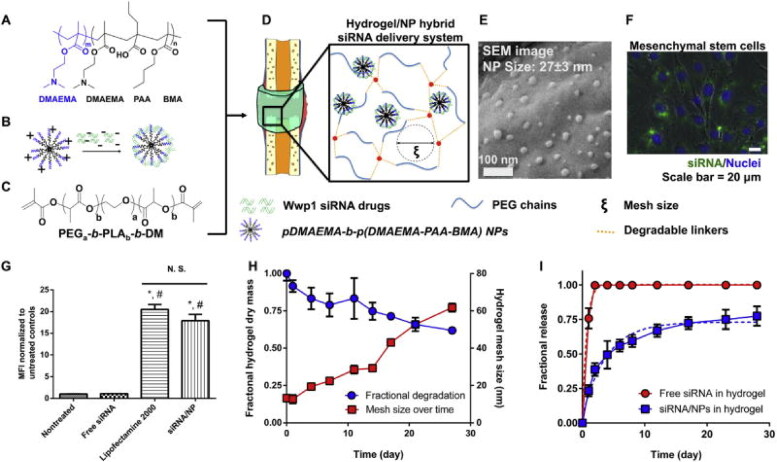
the hybrid nanoparticle (NPs)/Hydrogel siRNA delivery system delivers a platform for sustaining gene silencing. (A) siRNA transport diblock copolymers (B) Electrostatic interactions for forming complexes (C) The chemical structure of poly(ethylene glycol)-b-poly(lactide)-b-dimethacrylate (PEGa-b-PLAb-b-DM (D) siRNA/NP complexes (E) PEG hydrogels (F) FAM-labeled siRNA (G) MSCs loaded with free FAM-siRNA (H) PEG hydrogels during 4 weeks (I) siRNA/NPs and free siRNA loaded in PEG hydrogels. Reproduced with permission from (Wang et al., 2017).

They demonstrate the potential of a hybrid NPs/PEG hydrogel delivery method for regulating regeneration siRNA release that targets the WW domain-containing E3 ubiquitin protein ligase 1 (Wwp1) for wound repair in a clinically meaningful fractured model (as shown in Figure 7(A–E). Previous research has identified Wwp1 as a negative bone formation controller when there is an enhanced rate of bone growth and bone mass was detected in mice that lacked Wwp1. To form the siRNA/NP complexes (as shown in Figure 7(B)), Wwp1 siRNA was electrostatically combined with polymer diblock NPs (as shown in Figure 7(A)). These siRNA/NPs were then incorporated into hydrolytically degrade hydrogels developed from PEG-b-poly(lactide)-b-dimethacrylate (PEG-b-PLA-b-DM) (as shown in Figure 7(C)) that were intended to provide siRNA/NPs in a localized and sustained manner to fractures (as shown in Figure 7(D)). MSCs loaded with free FAM-siRNA had a higher median fluorescence intensity (MFI), Lipofectamine 2000 complexed with FAM-siRNA (positive control), or NPs was quantified and show the Swelling and breakdown of 4 kDa PEG hydrogels during 4 weeks (Figure 7(G,H)). Figure 7(I) shows the temporal release profile of siRNA/NPs and free siRNA loaded in PEG hydrogels.

Carbon nanotubes are utilized in tissue engineering due to their biological, structural as well as mechanical characteristics. They are also utilized as drug and gene transport vehicles (Yang et al., 2011). Sharmeen et al. (2018) described a multiwalled carbon nanotube-based drug-releasing matrix. The nanocomposite films containing multi-walled carbon nanotubes and gelatin-chitosan were developed and infused with ciprofloxacin, a typical antibiotic. The incorporation of carbon nanotubes into nanocomposite films resulted in enhanced drug release capability, as well as enhanced thermal and mechanical characteristics.

### Limitations and needs of bone tissue engineering

One of the most important issues in maxillofacial and orthopedic surgery is repairing significant bone deformities caused by trauma or disease (El-Ghannam, 2005). The composition of bone is 60–70% bone mineral (primarily hydroxyapatite [HA]), 10–20% collagen, and 9–20% water by weight (Wu et al., 2014). Autologous and allogeneic transplants are common therapeutic therapies for bone healing and growth, although they have several limits and risks, including morbidity in the donor area and immunogenic reaction (Hoexter, 2002). Tissue engineering has appeared as a potentially beneficial substitute to traditional transplants for the reconstruction of tissues and organs that have been lost or destroyed, overcoming their drawbacks (Salgado et al., 2013). Despite efforts to create innovative biomaterials that promote the body’s capacity to grow bone and connect with nearby bone tissue (Thibault et al., 2013), additional attempts are still necessary for biomaterials that have been designed to imitate biological bone tissue. To accomplish the need for bone tissue repair, these materials must be biocompatible, recyclable, porous, osteoconductive, integrated, and mechanically consistent with natural bone (El-Ghannam, 2005). Engineering Materials intended to imitate the biological and mechanical setting of genuine bone tissue matrix, as well as those that can help vascularize massive tissue constructions while preserving their physiological process, are a current challenge (Laurencin et al., 1999; Christenson et al., 2007; Stevens, 2008). Using biofunctionalization strategies, innovative biomimetic scaffolds must be designed to replicate topographical and biofactor cues at the nanoscale (Qu et al., 2015). Growth factors, for example bone morphogenetic proteins (BMP), and stem cells/osteogenic might be included into the biomaterials to encourage bone development, collagen production, and in vivo fracture healing (Reddi & Cunningham, 1990; de Guzman et al., 2013).

### Diagnosis

To efficiently diagnose and monitor orthopedic therapy for bone-related disorders like Paget’s syndrome, osteoporosis, and renal osteodystrophy in their early stages, it is crucial to detect the healing sites using versatile instruments (Yun et al., 2009). wireless implantable biosensors have currently been created for this purpose (Yang & Webster, 2011). Because of their high electrical conductivity, mechanical robustness, and distinct chemical-biological properties, carbon nanotubes have been identified as a favorable component for the development of bone sensors (Lin et al., 2004; Newman et al., 2013). As bone regenerates in response to electrical conduction, the increased electrical conductivity of CNTs stimulates bone growth. Supronowicz et al. (2002), found that there was a 46% rise in osteoblast propagation due to the addition of MWCNTs to PLA nanocomposites, as well as a 300% increase in calcium generation when in vitro substrate was subjected to an alternating current.

For such a sensor design, it is noteworthy that cell reactions transmit and communicate a multitude of physical and chemical signals to generate precise chemicals and proteins inside certain tissues and organs. After 21 days, Thomas and Sirinrath (Sirivisoot & Webster, 2008) demonstrated that the protein redox might be improved on MWCNTs produced from a nanotubular Ti electrode that has been anodized to detect bone development, stimulate osteoblast propagation, and induce differentiation.

**Figure 8.**
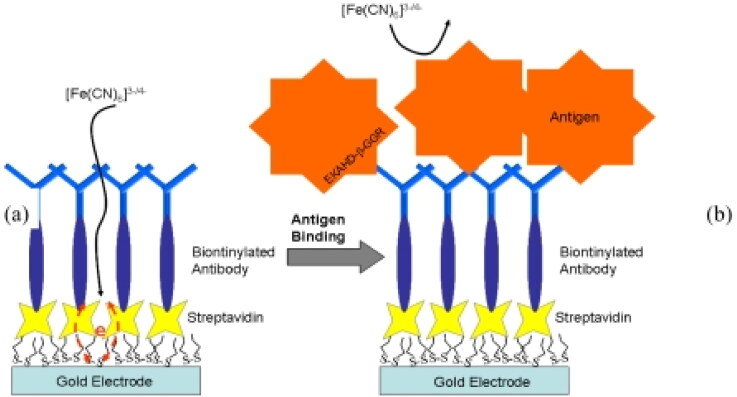
This Diagram depicts a label-free immunosensor designed to detect bone turnover markers. In part (a), a dithiodipropionic acid self-assembled monolayer is formed on a gold surface, followed by the immobilization of streptavidin as another self-assembled monolayer. Next, a biotinylated antibody is attached to the streptavidin. Part (b) shows how the antigen-antibody binding process occurs and how it disrupts the interfacial electron transfer reaction of [Fe(CN)_6_]^3−/4−^ (Yun et al., 2009).

Detecting bone-related degradation products is another method for monitoring bone turnover. Yun et al. (2009, 2007) developed an immunosensor for electrochemical impedance spectroscopy that does not require a label for sensing Type-1 collagen C-terminal teleopeptides (Figure 8). Such detecting approaches are built on the assumption that the bone remodeling cycle is divided into three stages: osteoclasts’ preexisting bone resorption, a phase of reversal marked on the bone surface by mononuclear cells, and the creation of new bone by osteoblasts to complete the voids left by resorption. These forms of detectors can also be fabricated using gold electrodes, but current developments propose employing carbon nanotube (CNT) electrodes for enhanced electrical conductivity and sensitivity. CNT-TiN nanocomposites, comprised of 12% CNTs by volume, demonstrated a 45 percent increase in electrical conductivity compared to TiN materials (Jiang & Gao, 2006).

In the orthopedic discipline, mixing medication delivery with implanted wireless sensors allows a radio frequency-controlled diffusion-controlled drug delivery system on demand (Yang & Webster, 2011). Employing nanotechnology, future sensors will be able to detect new bone development and, if it is not occurring, discharge medicines to increase new bone formation.

The extraordinary regenerative potential of hMSC-based stem cell therapies makes them the favorite choice for bone repair and fracture restoration (Wimpenny et al., 2012; Bull et al., 2014). To keep track of these procedures, hMSCs were tagged with various NPs, including superparamagnetic iron oxide (SPIO) NPs, quantum dots (QDs), gold NPs, and fluorescence-labeled mesoporous silica NPs (Tautzenberger et al., 2012). The fluorescent tagging of osteoblast cells with HA produced with the nucleating seed of hydrophilic CdSe/ZnS QDs enables real-time confocal imaging of the cell. A research study conducted on MC3T3-E1 osteoblast cells revealed that the cells should ingest HA with surface-tailored QDs displaying fluorescent patches in the cytosol, but not QDs and HA NPs. Remarkably, only the cytoplasm of MC3T3-E1 osteoblast cells displayed fluorescence (Hsieh et al., 2009). Several studies have looked at the usage of gold nanoparticles as agents of contrast in micro-CT applications (Popovtzer et al., 2008; Xi et al., 2012) and enhanced X-ray imaging technologies (Ahn et al., 2013) Because gold has a greater absorption coefficient than iodine and less interaction with tissues and bones, gold NPs enhanced contrast at lower dosages while extending imaging time (Ahn et al., 2013) SPIO NPs are also intriguing materials for use as MRI contrast agents. Nonetheless, It must be remembered that bone is a powerful target organ that presents a unique challenge for cell labeling (because of its significant mineralization grade), making tagged cell visibility in MRI challenging (Tautzenberger et al., 2012). In a current investigation, electrospinning was used to incorporate a multifunctional contrast agent composed of HA nanocrystals into a PCL nanofibrous scaffold. This initial investigation was conducted to ultimately utilize the MR contrast imaging capacity using nanofibrous frameworks for imaging changes in real-time within the tissue-engineered structure (Ganesh et al., 2014) in a clinical study conducted on osteoblasts, SPIO NPs bound to RGD receptors stimulated cells for three weeks. After cyclical magnetic stimulation, tagged cells exhibited increased osteopontin up regulation and osteo-related protein manufacturing. These findings suggest that the co-culture of SPIONs and osteoblasts does not prevent osteogenic activity. In addition, SPIONs may be attached to specific membrane receptors before being subjected to fluctuating magnetic fields in a magnetic force bioreactor to excite labeled receptors and deliver mechanotransduction across the cell membrane (Wimpenny et al., 2012).

Related efforts are being made to reduce infection and inflammation of orthopedic implants. As stated in the preceding section, graft- related infections pose a grave risk to patient health, and their therapeutic care is expensive. These infections are not easy to recognize to diagnose because of the formation of biofilms, intracellular bacteria, and posttraumatic and aseptic alterations. There is a clinical need for diagnostic instruments that can identify pathogenic infection forms. Utilizing the benefits of NPs as contrast agents in MRI and CT imaging, Nanotechnology has the potential to be a vital diagnostic and eradicating instrument for infections. Additionally, nuclear medicine, which uses radiolabeled infection tracers, appears to be a potential treatment for implant-associated infection diagnosis in clinical settings. Rapid progress is being made in the creation of multiplexed imaging modalities as well as the detection of particular infection tools (Potapova, 2013).

Key parameters that can be used to characterize implant-bone interface infections include biological characteristics like temperature and pH levels near the implant’s border (Potapova, 2013). They might transmit pH data via mimicked skin by adhering to a passively-powered radio frequency recognition device. This instrument displayed a Nernstian response across a broad pH range (2–12) and maintained sensitivity for 120 days. It is therefore anticipated that new advances in diagnostic imaging will substantially reduce the risk of implant-associated infections being incorrectly or tardily diagnosed.

Unlike MRI, several nanotechnology-based detection instruments are transforming the field of orthopedics. In osteoporosis cases, for instance, diagnostic techniques are crucial for transporting precise data quickly, affordably, and noninvasively. Before the development of nanomaterial-based techniques, there were very few reliable identification techniques (Garimella & Eltorai, 2017). Nanotechnology-based innovative technologies facilitate portable osteoporosis diagnosis. Particularly, a biochip has been developed as a consequence of research that uses gold NPs to detect an osteoporosis-related protein. It has been revealed to properly evaluate the quality of bone and accurately recognize and quantify degeneration of the bones (Garimella & Eltorai, 2017). In addition, using fluorescent probes to recognize NPs might aid in the assessment of tumor treatment response (Hennig et al., 2015). This method might offer a more accurate estimate of the histopathological evaluation after tumor removal than the remaining tumor volume (Young et al., 2012).

### Cancer therapy

Bone cancer-related skeletal complications are an essential health care issue. Osteosarcoma is the most typical form of bone tumor and the 3rd-leading cause of cancer in children and adolescents (Kansara & Thomas, 2007). The most prevalent region of cancer metastases is bone and is especially significant in prostate and breast malignancies due to the high prevalence of bone metastases in these diseases (Ovid’ko & Sheinerman, 2011). The most usual methods for treating bone metastases are radiotherapy, surgery, bisphosphonates, systemic chemotherapy, and radioisotopes (Suva et al., 2011). The latest developments have resulted in the creation of multifunctional bionanomaterials capable of targeting and delivering therapeutic medications or DNA to bone tumors (Mohamed et al., 2012). To induce hyperthermia, a variety of composite materials having differing amounts of magnetite are utilized (Li et al., 2006; Hu et al., 2006). Andronescu et al. (2010) manufactured a bone implant component and hyperthermia generator made of magnetite-enriched collagen/HA composite for the cure of cancer of the bone. Hu et al. (2006), fabricated a 3D nanomagnetite/CS rod that might be used to induce hyperthermia locally in bone lesions. In a separate investigation, Murakami et al., (2008), created a magnetite/HA composite that allows for direct adherence to bones via HA as well as heat production from magnetite (when subjected to an AC magnetic field) for bone cancer hyperthermia therapy. This composite had microporous dimensions of almost 400 m and submicroporous dimensions of about 0.2 m. Magnetite aggregates were shown to be firmly confined in rod-shaped HA particle cages at concentrations of 30 mass percent or less.

### Nanotoxicology

There have been reports regarding the potential dangers of NPs to human health and the surrounding, which might occur from exposures throughout their life cycle, as a result of the swiftly developing nanotechnology science and the creation of new items with a vast array of uses (Thomas & Sayre, 2005). In recent times, there has been a lot of interest in nanotoxicology, the study of the health concerns or adverse impact of manmade nanostructures on living organisms (Oberdörster et al., 2005). In practise, the variety of manufactured NPs and their endless possible applications have presented significant difficulties for safety evaluation. Analytical techniques for detecting and quantifying nanoscale material concentrations in the surroundings and the human body are also still in the beginning stages of growth (Majestic et al., 2010). Certain NPs can get inside the human body and move to multiple organs through the lymphatic and circulatory systems, according to human and animal studies (Bakand et al., 2012). Recently, harmful reactions to NPs produced by the deterioration of embedded nanomaterial, through wear debris from nanofeatured synthetic joints, and heavy metals (cobalt, nickel, and iron catalysts) persisting in CNTs have been documented (Zhang & Webster, 2009). Although nanophase materials have enhanced wear-failure characteristics, when exposed to physiological stress factors, detritus might emerge from the articulating parts of nanostructured orthopedic implants (Webster & Ahn, 2007). Possible adverse effects include asthma exacerbation, inflammation, metal particulate fever, chronic inflammatory lung disorders fibrosis, as well as carcinogenesis (Cheng et al., 2013). Due to oxidative stress, when delivered in moderate doses, silica NPs have been proven to have minimal toxicity in vivo. CNTs have also been observed to be deleterious to the lung and embryo (Li et al., 2012). Notably, very little is known regarding the fundamental toxicological processes accountable for potential NPs toxicity (Bakand et al., 2012). It is believed that the increased generation of ROS is the primary source of toxicity at the nanoscale material (Cheng et al., 2013). It has been demonstrated that the probability for toxicity rises as particle size decreases, whenever a similar material is used, such as carbon and titanium dioxide, is inert in bulkier form (Fan & Alexeeff, 2010). Consequently, the connections of nanomaterials with live organisms, along with the biological impacts of these components must be investigated in depth. Specifically, the connection between the characteristics of nanomaterials (surface area, shape, size, etc.) and their toxicity must be demonstrated (Fischer & Chan, 2007). In addition to technological advancements that expand the market for nanomedicine (Webster, 2006; Morigi et al., 2012). We assume that much more in vivo studies are required, toxicological studies, and clinical studies are required before NPs for orthopedic applications can be mass-produced commercially.

### Future perspective and concluding remarks

Nanobiomaterials can be used in orthopedics, according to preliminary research; however, additional improvement is essential for achieving practical application. The aim is to make bioactive bone regenerative scaffolds that can change typical tissues while interacting in relation to their environment, reacting to environmental variations, and actively influencing cellular processes to speed up bone development, shortened restoration time, and a quicker recovery to functioning. Future research will almost definitely concentrate on enhancing design methods using NPs and other fabrication methods. It is crucial to comprehend the molecular processes underlying cell-nano biomaterial connections. In addition, caution should be taken when proving nanomaterial biosafety and limiting their consequences. Concerns exist about the toxicity of NPs produced by wear and strain. At the nanoscale, metals react in different ways and have distinct material characteristics at the macroscale than at the microscale.

Therefore, traditional grafts with specific features treated using nanotechnology are favorable to transplants composed of NPs. This precludes the possibility of nanomaterials dispersing and influencing tissue toxicity. Regulation has been proposed in response to these issues. Because of the unknown therapeutic advantages and the risk of toxicity, and the high expense of nanostructured implants and prostheses, companies remain hesitant to develop them (Smith et al., 2018). Concerns exist about the toxicity of NPs produced by wear and strain. Metals react and exhibit different material characteristics at the nanoscale than at the microscale.

Although nanotechnology is still in its infancy, it can enhance orthopedic diagnosis, management, and research. The performance of the service and commercial industries sectors validates the notion that nanotechnology will play a crucial role in future curative treatment. Nanotechnology has the potential to significantly minimize the amount of various conventional medications and facilitate an abundance of inventive applications. Nanotechnology makes more accurate treatment approaches, as a result of which more effective and long-term implants, reduced infection control, and boost tendon and bone regeneration. Massive efforts are being made in basic science research and are beginning to realize the potential benefits of nanomedicine, particularly in orthopedics. Though, more study is needed to comprehend completely the protection and utility of this advanced technology.

## Data Availability

On reasonable request, all data can be obtained from the respective author.
